# CD34^+^ Cells Represent Highly Functional Endothelial Progenitor Cells in Murine Bone Marrow

**DOI:** 10.1371/journal.pone.0020219

**Published:** 2011-05-31

**Authors:** Junjie Yang, Masaaki Ii, Naosuke Kamei, Cantas Alev, Sang-Mo Kwon, Atsuhiko Kawamoto, Hiroshi Akimaru, Haruchika Masuda, Yoshiki Sawa, Takayuki Asahara

**Affiliations:** 1 Group of Vascular Regeneration Research, Institute of Biomedical Research and Innovation/RIKEN Center for Developmental Biology, Kobe, Japan; 2 Division of Cardiovascular Surgery, Department of Surgery, Osaka University Graduate School of Medicine, Osaka, Japan; 3 Group of Translational Stem Cell Research, Department of Pharmacology, Osaka Medical College, Osaka, Japan; 4 Department of Orthopedic Surgery, Graduate School of Biomedical Sciences, Hiroshima University, Hiroshima, Japan; 5 Laboratory for Early Embryogenesis, RIKEN Center for Developmental Biology, Kobe, Japan; 6 Department of Biomedical Science, CHA Stem Cell Institute, CHA University, Seoul, Korea; 7 Department of Regenerative Medicine, Tokai University School of Medicine, Kanagawa, Japan; Istituto Dermopatico dell'Immacolata, Italy

## Abstract

**Background:**

Endothelial progenitor cells (EPCs) were shown to have angiogenic potential contributing to neovascularization. However, a clear definition of mouse EPCs by cell surface markers still remains elusive. We hypothesized that CD34 could be used for identification and isolation of functional EPCs from mouse bone marrow.

**Methodology/Principal Findings:**

CD34^+^ cells, c-Kit^+^/Sca-1^+^/Lin^−^ (KSL) cells, c-Kit^+^/Lin^−^ (KL) cells and Sca-1^+^/Lin^−^ (SL) cells were isolated from mouse bone marrow mononuclear cells (BMMNCs) using fluorescent activated cell sorting. EPC colony forming capacity and differentiation capacity into endothelial lineage were examined in the cells. Although CD34^+^ cells showed the lowest EPC colony forming activity, CD34^+^ cells exhibited under endothelial culture conditions a more adherent phenotype compared with the others, demonstrating the highest mRNA expression levels of endothelial markers vWF, VE-cadherin, and Flk-1. Furthermore, a dramatic increase in immediate recruitment of cells to the myocardium following myocardial infarction and systemic cell injection was observed for CD34^+^ cells comparing with others, which could be explained by the highest mRNA expression levels of key homing-related molecules Integrin β2 and CXCR4 in CD34^+^ cells. Cell retention and incorporation into the vasculature of the ischemic myocardium was also markedly increased in the CD34^+^ cell-injected group, giving a possible explanation for significant reduction in fibrosis area, significant increase in neovascularization and the best cardiac functional recovery in this group in comparison with the others.

**Conclusion:**

These findings suggest that mouse CD34^+^ cells may represent a functional EPC population in bone marrow, which could benefit the investigation of therapeutic EPC biology.

## Introduction

Since endothelial progenitor cells were shown to contribute to tissue vascularization after ischemic events in limbs, retina and myocardium [1], [2], EPC therapy has been studied as a new strategy in regenerative medicine. Rapid revascularization of injured and ischemic organs is essential to restore organ function. Thus EPC therapy depends largely on the functional activity of EPCs. The usage of EPC populations having different properties resulted in the existing controversial findings of EPC therapy [3], [4].

CD34 is a 105- to 120-kD transmembrane cell surface glycoprotein, which is selectively expressed within the human and murine hematopoietic systems on stem and progenitor cells [5], [6], [7]. It is also expressed in vascular endothelial cells. Human CD34^+^ endothelial progenitor cells have been widely used for animal experiments and clinical use [8], [9], [10], [11], [12]. However, little research has been conducted to identify mouse bone marrow-derived CD34^+^ cells regarding their angiogenic properties. Whereas c-Kit^+^/Sca-1^+^/Lin^−^ cells have been widely used as mouse endothelial progenitor cells [13], [14], [15], [16]. In our preliminary studies, we found that key homing-related molecules, Integrin β2 and CXCR4, were higher expressed in bone marrow CD34^+^ cells rather than in other commonly used c-Kit^+^/Sca-1^+^/Lin^−^ cells (KSL), c-kit^+^/Lin^−^ cells (KL) [17], Sca-1^+^/Lin^−^ cells (SL) [2].

Integrins are crucial transmembrane molecules that mediate cell adhesion, migration, and the homing of progenitor cells such as EPCs to ischemic tissue, possibly through the enhanced angiogenesis by homing stem cells [18]. The β2-integrins are involved in the homing of EPCs to the site of ischemia and are essential for their neovascularization capacity in vivo [19]. The activation of β2-integrin on EPCs has been shown to significantly improve the neovascularization capacity in vivo in a model of hindlimb ischemia [20]. CXCR4 is also crucial for homing of transplanted EPC into ischemic tissues. CXCR4 blockade is associated with an impaired incorporation of EPC into sites of ischemia-induced neovascularization and disturbed restoration of blood flow to ischemic limbs, suggesting that CXCR4 is important for therapeutic integration of EPC into the vascular bed [21].

Based on the above concerns and the mechanistic findings, we sought to identify a functional mouse EPC population via enhanced homing mechanism. In pursuit of this goal, we analyzed the EPC properties of mouse bone marrow derived c-Kit^+^/Sca-1^+^/Lin^−^ cells (KSL), c-kit^+^/Lin^−^ cells (KL), Sca-1^+^/Lin^−^ cells (SL) and together with CD34^+^ cells. Our results suggest that mouse CD34^+^ cells may represent a functional EPC population in mouse bone marrow.

## Results

### Population of KSL, KL, SL and CD34^+^ cells

**Figure 1:**
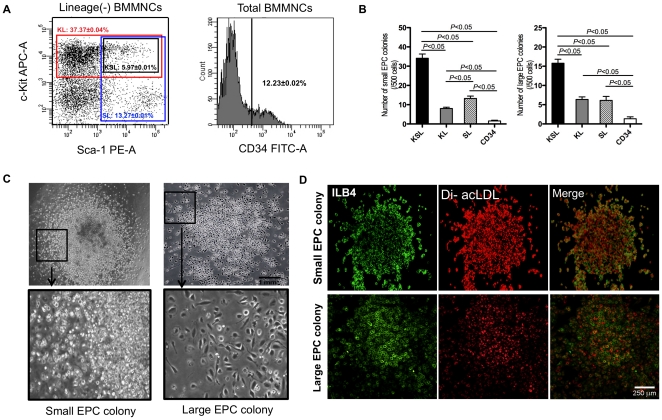
Isolation of KSL, KL, SL, and CD34^+^ cells by FACS and EPC-CFA. a, Lineage depleted BMMNCs were stained with APC anti-c-kit and PE anti-Sca-1 antibodies followed by FACS sorting. The cell fractions gated with black, red, and blue frames were defined as KSL, KL, and SL cells, respectively. Also BMMNCs were stained with a FITC anti-CD34 antibody followed by FACS sorting for CD34 positive cells. b, The number of colonies was counted in each group. Small EPC colony count in each group and large EPC colony count in each group. c, EPC-CFA was performed with KSL, KL, SL, and CD34^+^ cells, and the morphologies of small EPC colony and large EPC colony were dispalyed. The framed areas were magnified in lower panels. d, Representative double staining for Fluorescein isolectin B4 (ILB4) and DiI-acLDL in KSL cell-formed colonies. All assays were triplicated and demonstrated similar results.

Initially we determined the populations to investigate. For KL, SL and KSL cell isolation, lineage positive cells, counting about 90%, were depleted from total BMMNCs. KL cells and SL cells counted 37.37±0.04% and 13.27±0.01% respectively in lineage negative BMMNCs. KSL cells were included in KL or SL cells, and counted 5.97±0.01% in lineage negative BMMNCs. For CD34^+^ cell isolation, CD34^+^ cells were 12.23±0.02% in total BMMNCs (Figure 1a). The levels of expression of CD34 by KSL, KL and SL cells are 89.8%, 72% and 55.9%, respectively (Figure S1).

In summary, c-Kit^+^/Lin^−^ (KL, 3.737±0.004%) cells, Sca-1^+^/Lin^−^ (SL, 1.327±0.001%) cells, Sca-1^+^/c-Kit^+^/Lin^−^ (KSL, 0.597±0.001%) cells and CD34^+^ cells (12.23±0.02%) were isolated from mouse total BMMNCs by FACS.

### Low colony forming activity in CD34^+^ cells

First, we evaluated the EPC colony forming capacities of KSL, KL, SL and CD34^+^ cells as described before [16], [22]. An EPC-colony forming assay (CFA) was recently established in our laboratory. EPCs can form two types of EPC colony clusters, small (primitive) and large (definitive) EPC colonies. Small EPC colonies contain mainly small and round cells, whereas large EPC colonies are composed of large and spindle-shaped cells (Figure 1c). Both colony types are positive for the uptake of Ac-LDL and for labeling with an EC-specific marker, isolectin B4 (Figure 1d). In the current study, KSL cells had the highest capacity to form both large and small colonies (small colonies: 34±7/500 cells, large colonies: 16±3/500 cells) (Figure 1b). KL (small colonies: 8±2/500 cells, large colonies: 6±2/500 cells) and SL cells (small colonies: 13±4/500 cells, large colonies: 6±3/500 cells) formed fewer colonies than KSL cells but still overall more colonies than CD34^+^ cells. CD34^+^ cells showed the lowest colony forming capacity (small colonies: 2±1/500 cells, large colonies: 1±1/500 cells) (Figure 1b).

### Phenotypes of large and small EPC colonies

Small and large EPC colonies were collected separately after 7 days culture in methylcellulose medium. The phenotypes were analyzed by flow cytometry. The positive percentages of VE-cadherin, Flk-1 and Tie-2 were 5.3%, 3.7%, 2.9% in large colonies, respectively, 5.1%, 0.5%, 0.3% in small colonies, respectively (Figure S2a and S2b); while the positive percentages of c-Kit and Sca-1 were 4.8%, 4.4% in large colonies, respectively, 9.2%, 13.1% in small colonies, respectively (Figure S2a and S2b), which indicates that large colonies are more differentiated than small colonies. The positive percentages of CD19, CD3, CD14, CD41 and CD45 were 2.5%, 3.2%, 1.4%, 7.5%, 99.9% in large colonies, respectively, 0.1%, 0.2%, 0.3%, 9.1%, 100% in small colonies, respectively (Figure S2a and S2b).

### Enhanced endothelial phenotype of CD34^+^ cells following an adhesive culture assay

**Figure 2:**
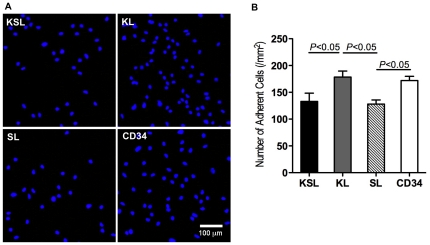
Culture assay with KSL, KL, SL, and CD34^+^ cells. Freshly isolated KSL, KL, SL, and CD34^+^ cells were cultured in 20%FBS/EGM-2MV medium on vitronectin-coated 4 well-chamber slides for 7 days. a, The cells were washed by PBS for three times and stained with DAPI. b, The numbers of adherent cells were counted and analyzed (n = 3). The assay was triplicated and demonstrated similar results.

The cell culture assay was performed to further characterize all cell populations. Cells were cultured in differentiation medium for 7 days. CD34^+^ (172±19 cells/mm^2^) and KL cells (179±28 cells/mm^2^) showed more numbers of adherent cells than KSL (133±38 cells/mm^2^) and SL cells (128±19 cells/mm^2^) (*P*<0.05, CD34 vs SL; *P*<0.05, KL vs KSL and SL) (Figure 2a and 2b).

**Figure 3:**
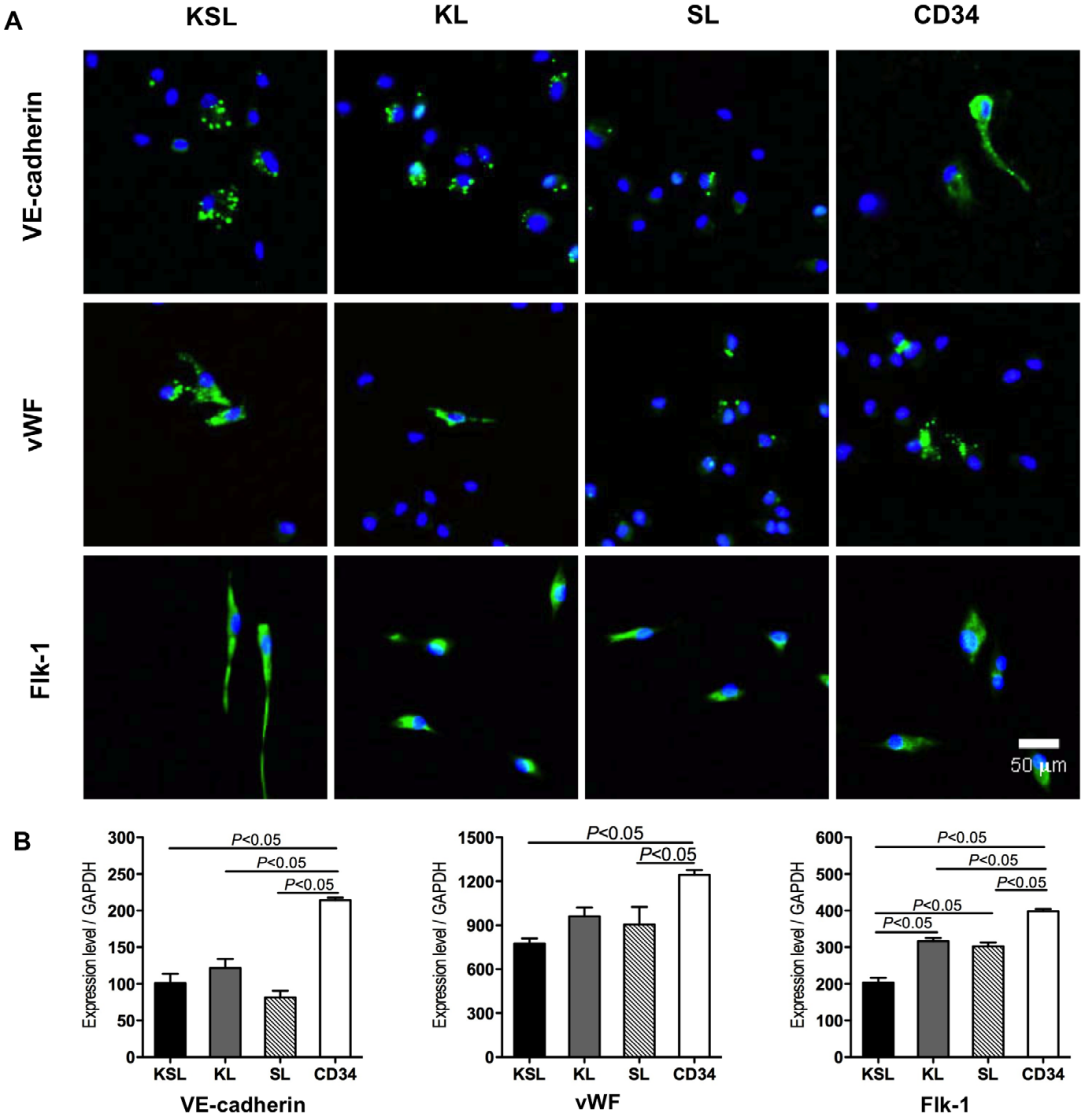
Assessment of endothelial markers in cultured KSL, KL, SL, and CD34^+^ cells by immunocytostaining and real-time RT-PCR. Freshly isolated cells were cultured in 20%FBS/EGM-2MV medium on vitronectin-coated 4 well-chamber slides for 7 days. a, Adherent cells were stained with endothelial markers, VE-cadherin, vWF, and Flk-1. b, mRNA expression levels of the markers in 3-days cultured cells were assessed by quantitative real-time RT-PCR. The mRNA expressions were normalized to GAPDH (n = 3). All assays were triplicated and demonstrated similar results.

The expression of endothelial markers in cultured KSL, KL, SL, and CD34^+^ cells was assessed by fluorescent immunocytochemical staining and real-time RT-PCR. Consistent with the previous report that cultured EPCs express endothelial markers such as VE-cadherin, vWF and Flk-1 [23], a significant number of cells expressed VE-cadherin, vWF and Flk-1 protein in each group (Figure 3a). RT-PCR revealed that at day three CD34^+^ cells had the highest mRNA expression levels of VE-cadherin, Flk-1 and vWF (Figure 3b). While all the cells before culture expressed low and similar levels of these markers (data not shown).

### Higher angiogenic capacity in CD34^+^ cells in vitro

**Figure 4:**
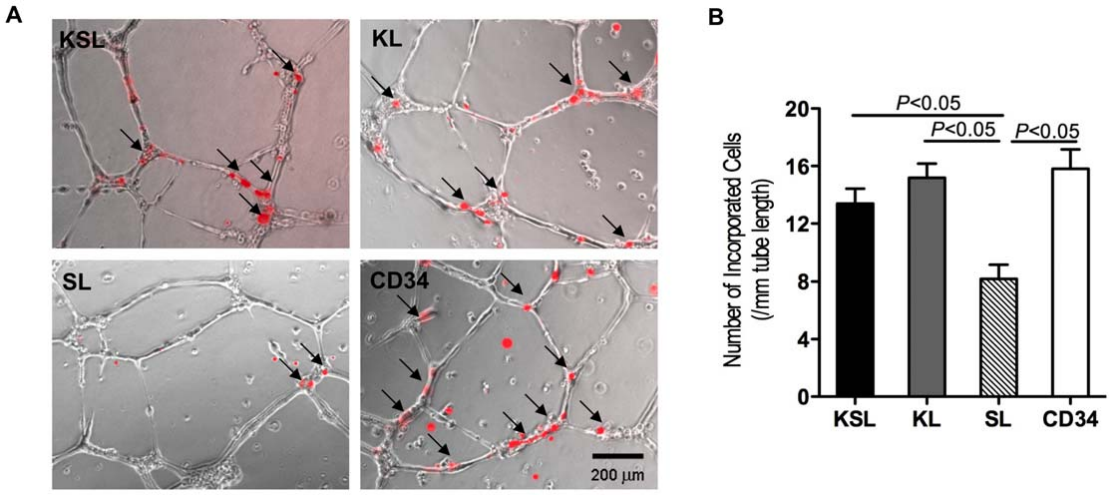
Tube formation assay by HUVECs incorporated with KSL, KL, SL, and CD34^+^ cells. KSL, KL, SL, and CD34^+^ cells were isolated via FACS and stained with DiI. DiI-labeled cells and HUVECs were seeded onto Matrigel-coated 96 well plates in 1% FBS/EBM2-MV without growth factors. After 24 hours in culture, incorporation of each cell population into tube-like structures formed with HUVECs was evaluated under fluorescence microscopy. a, Incorporated DiI positive cells were indicated by arrows. b, Number of incorporated DiI positive cells into tube-like structures was counted and averaged. All assays were triplicated and demonstrated similar results.

The in vitro incorporation capacity of the cells was assayed via a previously described tube formation assay system [24]. HUVECs were employed to form tube-like structures. KSL, KL, SL and CD34^+^ cells were seeded together with HUVECs, incorporating into tube-like structures and supporting their growth. One thousand (1,000) DiI-labeled cells and 12,500 HUVECs were seeded onto Matrigel-coated 96 well plates. After 24 hours in culture, incorporation of each cell population into tube-like structures formed by HUVECs was evaluated under fluorescence microscopy. Augmented cell incorporation was found for the CD34^+^, KSL and KL groups compared to the SL group (CD34: 16±3, KSL: 13±2, KL: 15±2, SL: 8±2; *P*<0.05, SL vs CD34, KL and KSL) (Figure 4a and 4b).

### Higher recruitment capacity of CD34^+^ cells in vitro and in vivo

**Figure 5:**
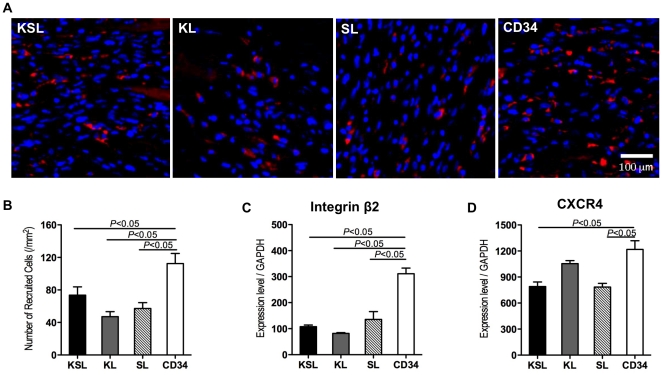
Assessment of cell recruitment and detection of homing molecules in KSL, KL, SL, and CD34^+^ cells. KSL, KL, SL, and CD34^+^ cells were freshly isolated by FACS and stained with DiI. DiI-labeled cells were systemically injected into mice three days after MI induction, and heart samples were examined histologically one day after cell injection. a, DiI positive cells (red) detected in ischemic myocardium under a fluorescence microscope. b, Number of DiI positive cells was counted in the ischemic border zone (bilateral sides of peri-infarct area) and averaged (n = 3). c and d, KSL, KL, SL, and CD34^+^ cells were freshly isolated by FACS, and Integrin β2 and CXCR4 gene expression levels of these cells were examined by quantitative real-time RT-PCR. All assays were triplicated and demonstrated similar results.

Next, we examined the homing capacity of these cells in mouse MI models. For tracking the fate of grafted EPCs in mouse myocardium, transplanted cells were labeled with CM-DiI after isolation by FACS and then delivered systemically three days after MI induction. Heart samples harvested one day after cell injection and examined for the presence of fluorescent-labeled cells. DiI-positive cells were observed in the infarcted myocardium of all examined groups (Figure 5a). However, the most dramatic immediate cell recruitment to ischemic myocardium was detected in the CD34^+^ cell-injected group (CD34: 112±41/mm^2^, KSL: 74±34/mm^2^, KL: 47±21/mm^2^ and SL: 57±24/mm^2^; *P*<0.05, CD34 vs KSL, KL and SL) (Figure 5b). No significant differences among the numbers of recruited cells in the KSL, KL and SL groups could be observed. Integrin β2 has been reported previously to be critical adhesion molecules for the homing of EPCs to ischemic tissue [18], [19], [20], [25]. SDF1/CXCR4 axis has also been shown to be one of the major chemokine/receptor signalings for EPC recruitment [26], [27], [28]. CXCR4 is also crucial for homing of transplanted EPC into ischemic tissues [21]. We thus assessed the mRNA expression levels of these homing-related molecules in the cells that were used for transplantation. We observed a marked up-regulation of integrin β2 and CXCR4 in CD34^+^ cells (Figure 5c and 5d). KL cells also exhibited higher expression levels of CXCR4 (Figure 5d).

### Enhanced cell retention, vasculogenic activity and reduced fibrosis in ischemic hearts by CD34^+^ transplantation

**Figure 6:**
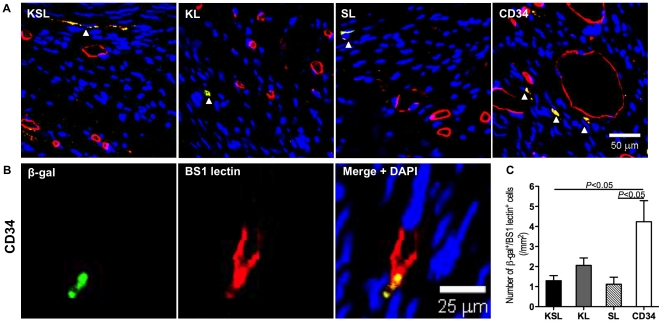
Assessment of cell incorporation into the neovasculature of the ischemic myocardium. KSL, KL, SL, and CD34^+^ cells were isolated by FACS from GtRosa26 transgenic mice expressing LacZ gene universally, and were systemically injected three days after MI induction. Heart samples were harvested following BS1 lectin systemic perfusion 28 days after cell injection. a, Recruited cells visualized by immunofluorescent staining for β-gal (green) and BS1 lectin perfused capillaries (red) were shown in all groups. Recruited cells co-localizing with capillaries were marked by arrowheads and shown in yellow. b, A representative confocal image from the CD34^+^ cell-injected group. c, The numbers of recruited cells that co-localized with capillaries were counted under a fluorescence microscope separately and averaged.

To track the injected cells in vivo for 28 days, we employed cells, isolated from ROSA mice, that constitutively express β-gal under the regulation of LacZ gene. Thus, cell differentiation and incorporation into the neovasculature of ischemic myocardium four weeks after surgery can be detected as β–gal^+^ cells by immunohistochemistry. Double fluorescent immunostaining of ischemic myocardium 28 days after surgery revealed that β-gal-expressing EPCs (green) were frequently observed in ischemic border zone and co-localized with vessels perfused with BS-1 lectin (red) in the CD34^+^ cell-injected group (Figure 6a). In contrast, a few KSL, KL and SL cells were observed in the ischemic border zone and incorporated into vessels (Figure 6a). Quantitative analysis for cell retention demonstrated that the number of incorporated cells into neovasculature was significantly higher in the CD34^+^ cell-injected group than those in the other groups (Figure 6c).

**Figure 7:**
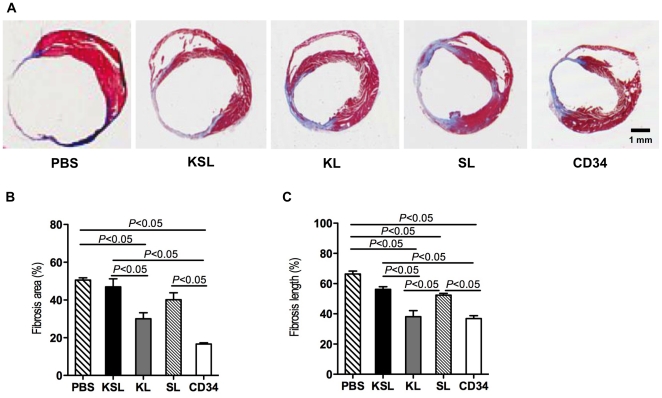
Histological analysis for MI size in ischemic myocardium. KSL, KL, SL, CD34^+^ cells and PBS control were systemically injected 3 days after MI induction. Heart samples were harvested following BS1 lectin systemic perfusion 28 days after cell injection. a, Heart sections were stained by Masson's Trichrome staining. Red indicates intact myocardium and blue indicates scared fibrosis area. b, The percent of fibrosis area in entire LV cross sectional area was calculated and averaged (n = 3). c, The percent of scar length in internal LV circumference was calculated and averaged (n = 3).

**Figure 8:**
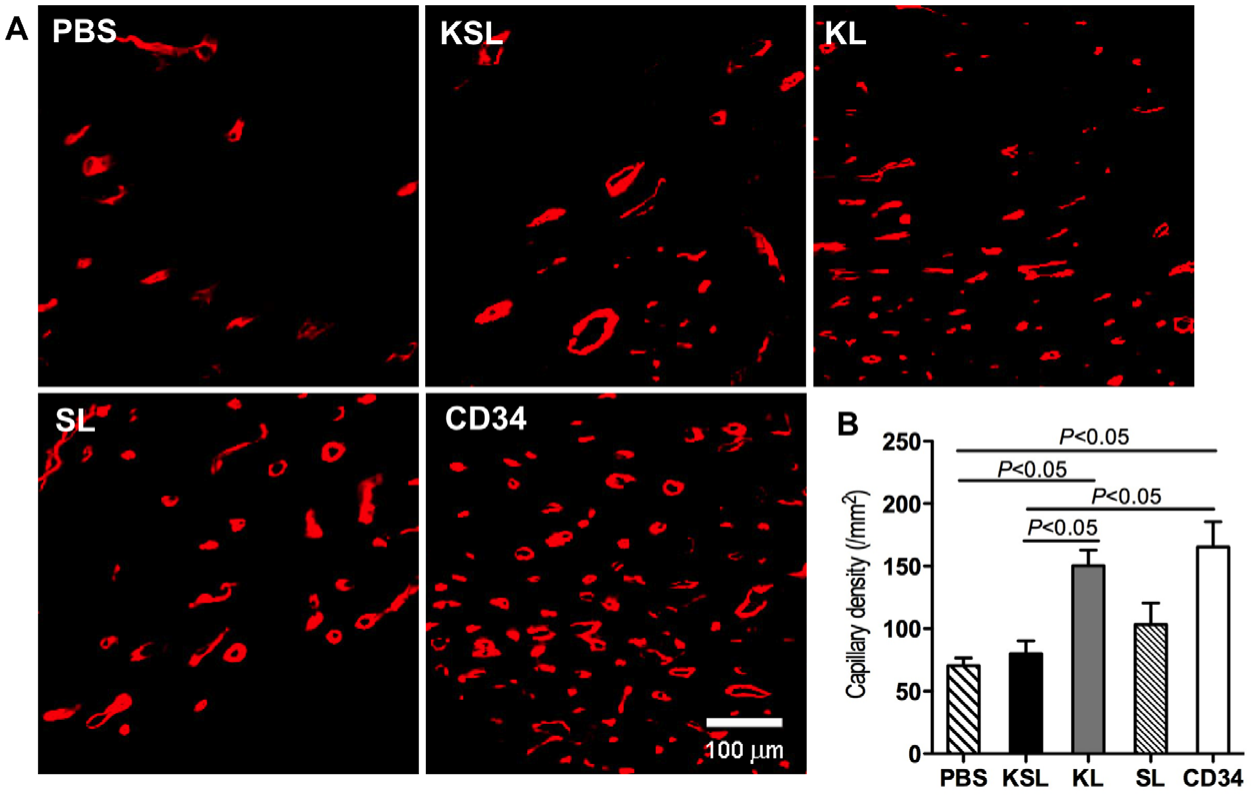
Histological analysis for capillary density in ischemic myocardium. KSL, KL, SL, CD34^+^ cells and PBS control were systemically injected 3 days after MI induction. Heart samples were harvested following BS1 lectin systemic perfusion 28 days after cell injection. a, Capillaries were visualized as tubular structure perfused by BS1 lectin. b, The numbers of capillaries were counted in bilateral sides of the peri-infarct zone on LV cross sections and averaged in each group (n = 3), respectively.

**Figure 9:**
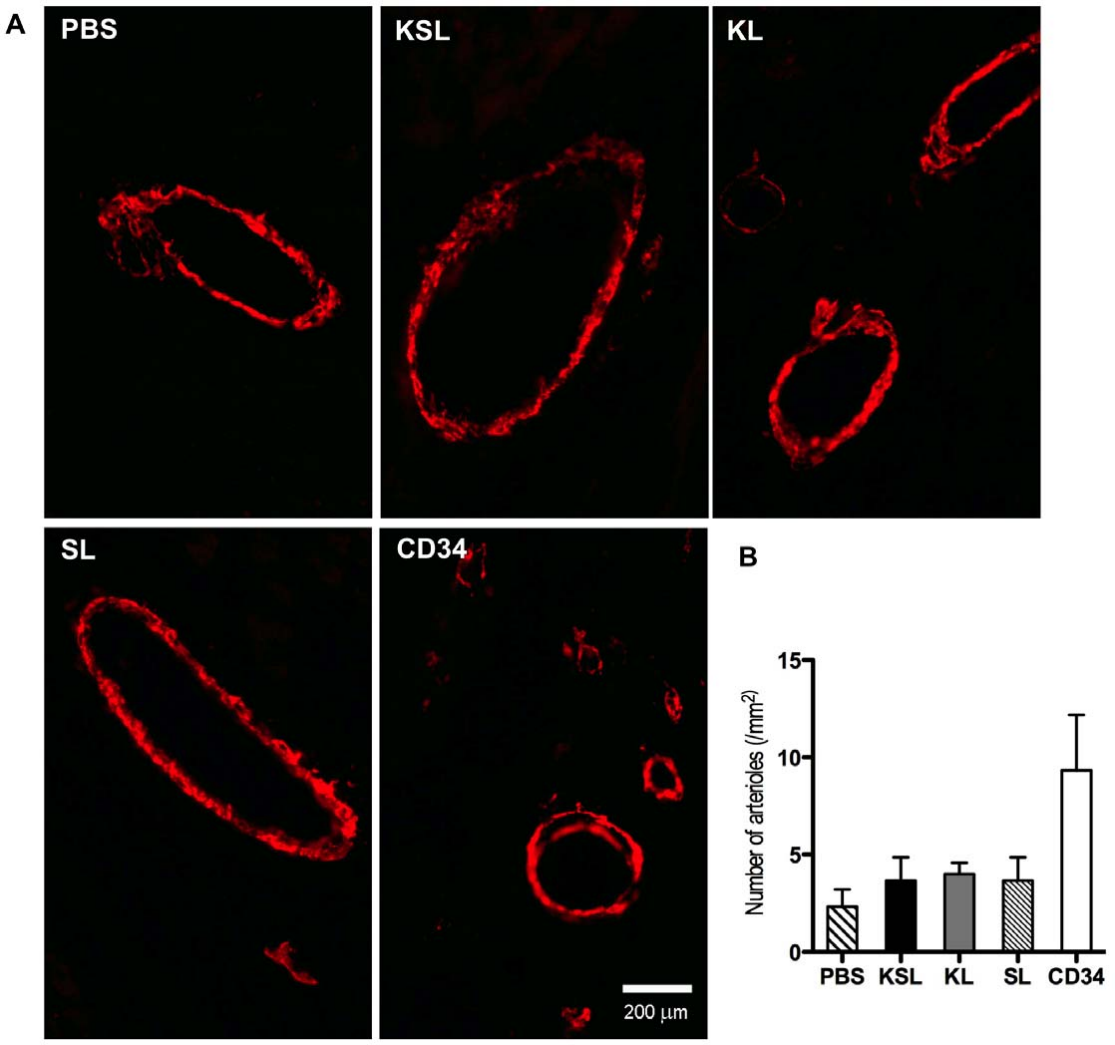
Histological analysis for arterioles in ischemic myocardium. KSL, KL, SL, CD34^+^ cells and PBS control were systemically injected 3 days after MI induction. Heart samples were harvested following BS1 lectin systemic perfusion 28 days after cell injection. a, Arterioles were visualized following α-smooth muscle actin staining. b, The numbers of arterioles were counted in bilateral sides of the peri-infarct zone on LV cross sections and averaged in each group (n = 3), respectively.

In order to evaluate the size of MI, Masson's trichrome staining was performed with heart sections at day 28. Representative images indicated that fibrosis area was stained in blue and intact myocardium was stained in red (Figure 7a). Quantitative analysis revealed that both the percent of fibrosis area in entire LV cross-sectional area and the percent of scar length in entire internal LV circumference were significantly reduced in the CD34^+^ cell-injected group compared with the KSL cell-injected group, the SL cell-injected group and PBS group (Figure 7b and 7c). The staining of in vivo-perfused BS1 lectin and α-smooth muscle actin reflects angiogenesis in functional vessels in the peri-infarct myocardium four weeks after MI in all groups (Figure 8a and 9a). The averaged capillary density in the bilateral ischemic border zones of LV, an index of neovascularization, was significantly greater in the CD34^+^ cell-injected group compared to the KSL cell-inject group and PBS group (CD34: 165±40/mm^2^, KL: 150±25/mm^2^, SL: 103±35/mm^2^, KSL: 80±21/mm^2^, PBS: 70±13/mm^2^; *P*<0.05, PBS vs CD34 and KL, KSL vs CD34 and KL) (Figure 8b). Although the averaged number of arterioles was higher in the CD34^+^ cell-injected group, no significant difference could be found between any two groups (Figure 9b). The KL cell-injected group also showed an increase in capillary density (*P*<0.05, KL vs KSL and PBS), reduced fibrosis area (*P*<0.05, KL vs KSL and PBS) and reduced fibrosis length (*P*<0.05, KL vs KSL, SL and PBS).

### Transplanted CD34^+^ cells effectively preserve LV function after MI

**Figure 10:**
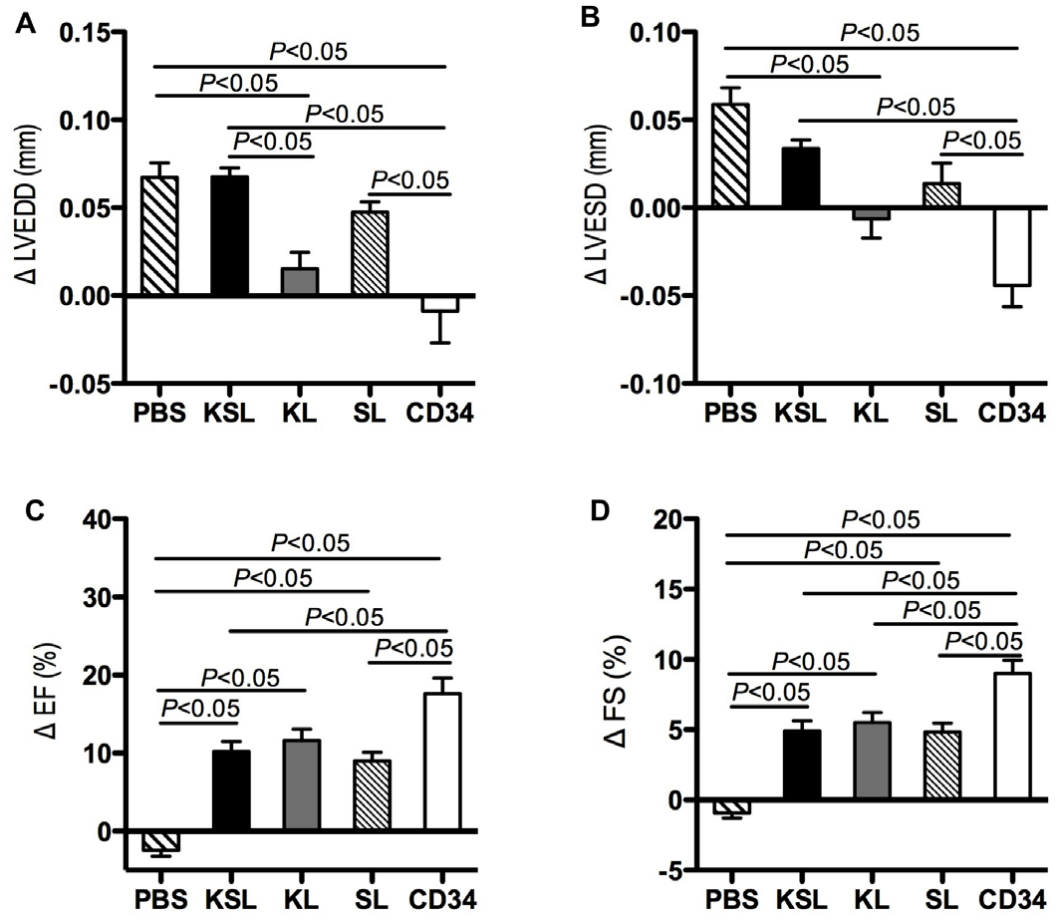
Functional analysis of treated hearts by echocardiography. Echocardiographic parameters for cardiac function 28 days vs. 7 days after myocardial infarction and treatments. The following parameters: left ventricular end diastolic diameter (LVEDD) (a), left ventricular end systolic diameter (LVESD) (b), left ventricular ejection fraction (EF) (c) and left ventricular fractional shortening (FS) (d) were measured in the PBS-, the KSL-, the KL-, the SL- and the CD34^+^ cell-injected groups, and change of each parameter between day 7 and day 28 after surgery was calculated and averaged (n = 3).

There were no significant differences in preoperative echocardiographic parameters, LVEDD (left ventricular end-diastolic dimension), LVESD (left ventricular end-systolic dimension), EF (ejection fraction) and FS (fractional shortening) among any groups. Echocardiography performed 7 days and 28 days after cell transplantation demonstrated that ΔLVEDD (day 28-day 7) was significantly smaller in the CD34^+^ cell-treated group than in the PBS-, the KSL- and the SL-treated groups (*P*<0.05 vs PBS, KSL and SL) (Figure 10a). The ΔLVEDD was also significantly smaller in the KL-treated group than in the PBS- and the KSL-treated groups (*P*<0.05) (Figure 10a). However, ΔLVEDD was similar in the PBS-, the KSL- and the SL-treated groups (Figure 10a). The ΔLVESD (day 28-day 7) was significantly smaller in the CD34^+^ cell-treated group than in the PBS-, the KSL- and the SL-treated groups (*P*<0.05 vs PBS, KSL and SL) (Figure 10b). The ΔEF (day 28-day 7) was significantly greater in the CD34^+^ cell-treated group than in the PBS-, the KSL- and the SL-treated groups (*P*<0.05 vs PBS, KSL and SL) (Figure 10c). The ΔEF was also significantly smaller in the PBS-treated group than in the KSL-, the KL- and the SL-treated groups (*P*<0.05) (Figure 10c). The ΔFS (day 28-day 7) was significantly greater in the CD34^+^ cell-treated group than in the PBS-, the KSL-, the KL- and the SL-treated groups (*P*<0.05 vs PBS, KSL, KL and SL) (Figure 10d). The ΔFS was also significantly smaller in the PBS-treated group than in the KSL-, the KL- and the SL-treated groups (*P*<0.05) (Figure 10d). Overall, transplantation of CD34^+^ cells exhibited significant LV functional recovery among all cell-transplanted groups following MI, which is consistent with the results of histological analysis.

## Discussion

The present study was designed to determine a functional population for murine EPC transplantation by systemic injection. The results indicated the CD34 could be a better marker for isolating functional EPC populations in mouse bone marrow compared with c-Kit, Sca-1 and their combination. We have demonstrated that mouse bone marrow-derived CD34^+^ cells exhibit characteristics of endothelial progenitors both in vitro and in vivo as follows: 1) CD34^+^ cells possess highly adhesive feature and highly express endothelial markers following the conversion to an adhesive cell phenotype despite of their less colony forming capacity, 2) CD34^+^ cells have a better homing capacity to ischemic tissue through adhesion molecule and chemokine/receptor-dependent mechanism, and 3) Recruited CD34^+^ cells are frequently incorporated into functional vessels contributing to the reduction of MI size, the enhanced angiogenesis and the significant LV functional recovery following MI.

Since phenotypic HSCs (c-Kit^+^/Sca-1^+^/Lin^−^ cells) [29] have been described to have the ability to give rise to endothelial progeny [30], [31], [32], KSL cells have been widely used as mouse EPCs [13], [14], [15], [16], displaying colony forming capacity, endothelial differentiation capacity and vasculogenic activity. In addition, Takahashi et al [2] used Sca-1^+^ cells from peripheral blood as an EPC-enriched population and demonstrated their differentiation into endothelial cells in mice with hindlimb ischemia. Cheng et al [17] purified CD31^+^/c-Kit^+^/Lin^−^ cells from bone marrow and confirmed their contribution to neovascularization following bone marrow transplantation. Very recently, Kim et al [33] has demonstrated high vasculogenic property of CD31 positive cells in BMMNCs. On the other hand, Balsam et al [34] and Murry et al [35] reported that mouse bone marrow HSCs isolated as c-Kit^+^/Lin^−^ cells or c-Kit^+^/Sca-1^+^/Thy1.1^lo^/Lin^−^ cells adopt mature haematopoietic fates in the infarcted myocardium. Given the controversy and also considering that CD34 expression patterns during early mouse development are related to modes of blood vessel formation [7], we analyzed the EPC properties of mouse bone marrow derived c-Kit^+^/Sca-1^+^/Lin^−^ cells (KSL), c-kit^+^/Lin^−^ cells (KL), Sca-1^+^/Lin^−^ cells (SL) and together with CD34^+^ cells. As for the adherent EPC formation, CD34^+^ cells and KL cells changed their phenotypes more frequently into adherent cells than the others. We also examined mRNA and protein expressions in CD34^+^, KSL, KL and SL cells cultured under an endothelial culture condition. Although immunocytochemistry revealed that VE-cadherin, vWF and Flk-1 were similarly positive in all types of cells, mRNA expression of these markers was greatest in CD34^+^ cells, suggesting that endothelial differentiation may occur more robustly in CD34^+^ cells than in the others. We also examined the expressions of CD45 and another EC marker CD146. CD45 and CD146 were expressed partially in the cells in each group (Figure S3), suggesting that the cells were partially differentiated exhibiting both hematopoietic and endothelial phenotypes. A few cells also expressed CD34 during the culture (Figure S3).

The cell homing capacity to ischemic tissue is also one of the unique characteristics in EPCs. The major distinct assortment of EPCs is proposed to lay in their *in vivo* phenotype, distinguishing “circulating EPCs” from “tissue EPCs”. Circulating EPCs can be isolated from PB and BM and represent a population of suspended non-adhesive cell phenotype. These cells migrate from their initial protective niches in the BM into the blood stream, and home via the circulatory system to sites undergoing vascular regeneration and repair, transforming into local adhesive tissue EPCs. Therefore, we further examined EPC groups regarding the biological properties for homing *in vitro* and the pathophysiological recruitment from circulation *in vivo* using a mouse MI model. The most dramatic cell recruitment to ischemic myocardium immediately after MI was observed in the CD34^+^ cell-injected group. Integrinβ2 and CXCR4 have been reported previously to be critical for the homing and neovascularization capacity of EPCs. They were significantly up-regulated in CD34^+^ cells compared to the others by RT-PCR analysis, providing a very possible explanation for the most striking cell recruitment of CD34^+^ cells to ischemic myocardium. In fact, we also assessed many angiogenic factors including VEGFs, Ang-1, IGF, PLGF and SCF in all types of cells, but the expression levels of all these genes were similar among them (data not shown). Therefore, the homing activity is one of the crucial mechanisms in regard to revascularization. With the most homing and recruited cells, the CD34^+^ cell-injected group performs the best. It is noteworthy that CD34^+^ cells and KL cells showed a similar adhesive capacity in vitro, while in vivo they didn't. We speculate that it is caused by the up-regulated expressions of integrinβ2 and CXCR4 in CD34^+^ cells. Furthermore, the highest number of cells was incorporated into vascular structure in the CD34^+^ cell-injected group in the 28 days follow up study, resulting in the reduced fibrosis, the increased vascularity and the significantly enhanced LV functional recovery in the ischemic myocardium. Another finding is that KL cell-injected group also showed greater capillary density and smaller fibrosis area/length than SL and KSL groups, possibly due to more abundant incorporation into neovasculature. Since myocardial regeneration requires vascular regeneration and cardiomyocyte regeneration as well, the recruited cells might differentiate into cardiomyocytes in addition to endothelial cells, as we observed for human CD34^+^ cells [36].

To make sure that vasculogenesis and the observed recruitment capacities of mouse bone marrow CD34^+^ cells indeed reflect the characteristics of EPCs rather than mature endothelial cells (ECs), we analyzed the presence and expression of several other cell surface markers such as CD31, VE-cadherin, Flk-1 and Tie-2 in the CD34^+^ cell population by FACS analysis (Figure S4). Positive percentages of CD31, VE-cadherin, Flk-1 and Tie-2 were 83%, 0.5%, 1.1% and 4%, respectively, indicating that no substantial EC contamination of CD34^+^ cells is present.

In the conventional EPC culture, two major cell types have been shown to emerge out of MNC cultures: EC-like cells (i.e., early EPC, cultured EPC) and endothelial outgrowth cells (i.e., EOCs) [37]. While EOCs are proved to derive from endothelial colony forming cells (ECFCs), EC-like cells have not been identified to develop from any colony forming unit, although CFU-EC was initially presumed as an origin of EC-like cells, but was later defined as an aggregate of hematopoietic cells and EPCs. Lately, a new EPC colony forming assay (EPC-CFA) has been developed, challenging some of the classical views predominant in the field and opening the possibility to illustrate the developmental hierarchy of EPCs [15], [16], [22], [38]. The methodological concept of EPC-CFA was recently introduced and further developed for the use of human EPCs (Masuda et al., revising in Circulation Research). In this research, small-EPCs are characterized as “primitive EPCs”, which are possibly derived from further immature and proliferative EPCs, and large-EPCs as “definitive EPCs” which are more prone to differentiation and promoting EPC-mediated cell functions required for vasculogenesis. The definitive EPCs are capable of differentiating into a non-colonizing large EPC phenotype, similar to “EC-like cells (i.e., early EPC, cultured EPC)” detected by conventional EPC culture [39], [40], [41]. Therefore, “EC-like cells” are speculated to represent further differentiating EPCs, departed from the niche of colony forming EPCs such as small- and large-EPCs. In contrast, EOCs demonstrate very similar expressional profiles and biological functions to differentiated ECs such as human umbilical vein ECs and human microvascular ECs, and are considered to be generated from different stem cell fraction compared to EC-like cells as previously reported [42], [43], [44]. Later studies by Case et al or Timmermans et al demonstrated that CD34^+^CD133^+^VEGFR-2^+^ or CD133^+^ cells are hematopoietic progenitor cells that do not yield EOCs [44], [45]. These findings support the idea that EOCs are not likely to derive from hematopoietic stem cells (HSCs) or hematopoietic progenitor cells (HPCs) originating from the BM, while EC-like cells and two types of colonies in EPC-CFA are relating to HSCs or HPCs. Therefore, the primary and original phenotype of EPC-CFA and EC-like cells seems different from EOCs (ECFCs), though it is not conclusive due to the immaturity in respective EPC biology.

**Figure 11:**
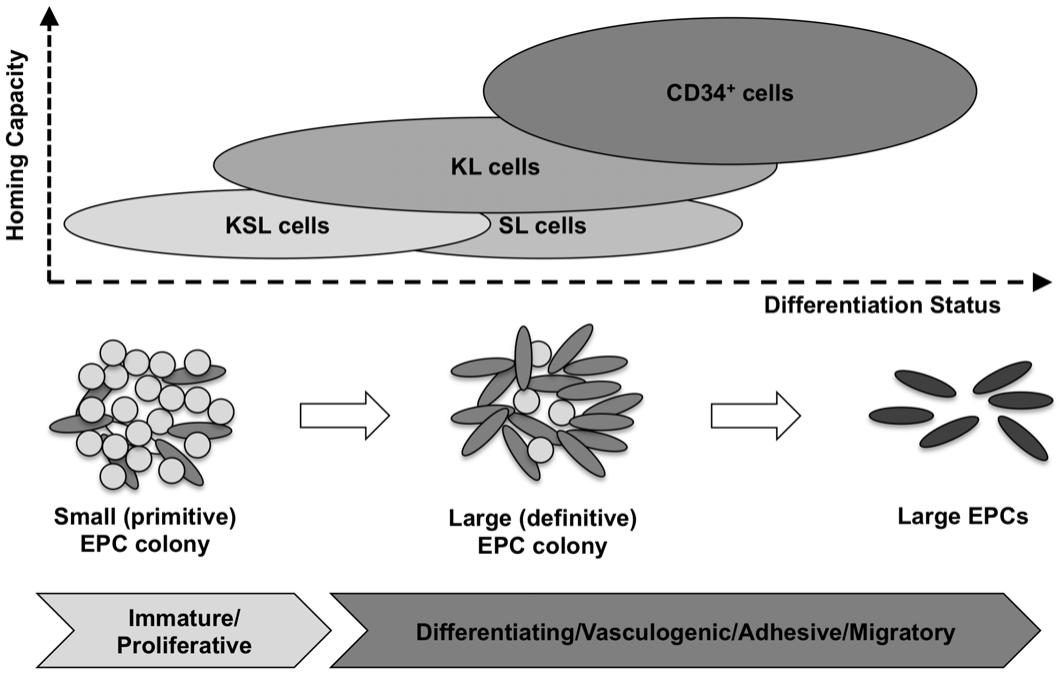
Stem/progenitor differentiation hierarchy of KSL, KL, SL and CD34^+^ cells. In EPC-CFA, small EPC colonies are capable of differentiating into large colonies and large EPC colonies can further differentiate into adherent, more vasculogenic non-colony forming EPCs in culture. Small colonies reveal a predominant potential for proliferation, while large colonies demonstrate a predominantly vasculogenic potential including cell adhesion and tube-like structure formation in vitro (unpublished data, lower panel). Based on the EPC colony numbers of KSL, KL, SL and CD34^+^ cells, the conceivable extent of homing capacity (Y-axis) and differentiation status (X-axis) in each cell type is indicated in the upper panel.

In terms of EPC colony forming activity, KSL cells formed a substantially greater number of small (primitive) EPC colonies and large (definitive) EPC colonies, especially small EPC colony forming activity of KSL cells was robust compared to the others, which is consistent with our previous data [16]. Both KL cells and SL cells showed a less level of small/large colony forming activity, while CD34^+^ cells demonstrated a further low activity. In the cell culture assay and in vivo assays, CD34^+^ cells had the highest activity. It seems to be controversial that CD34^+^ cells are the most functional cells, but they can only form a few colonies. In fact, it is not surprising and very reasonable. Because CD34^+^ cells count 12.23±0.02% in BMMNCs, while KL cells count 3.737±0.004%, SL cells count 1.327±0.001%, KSL count 0.597±0.001%, respectively, which indicates CD34^+^ cells are partially differentiated cells. In EPC differentiation hierarchy, it was confirmed that small EPC colonies are capable of differentiation into large EPC colonies and that large colonies can further differentiate into adherent, more vasculogenic EPCs in culture (unpublished data by Masuda et al) (Figure 11). In addition, small EPC colonies have a predominant stem cell-like potential for proliferation and large EPC colonies possess a predominantly vasculogenic potential including cell adhesion, tube formation activity in vitro and contribution to de novo blood vessel formation in ischemic tissue (Kwon et al [16], unpublished data by Masuda et al and Tsukada et al and Figure S2) (Figure 11). Taken the EPC differentiation and in vivo data together, our results suggest that KSL cells are most immature, while CD34^+^ cells are most differentiating population in mouse EPCs that we studied (Figure 11). Therefore we hypothesize the possible differentiation hierarchy of KSL, KL, SL and CD34^+^ cells (Figure 11).

Consequently, our data demonstrates that CD34 may be a better and more suitable marker for mouse functional EPCs with higher homing and vasculogenic properties, which could benefit the investigation of therapeutic EPC biology. Assuming the heterogeneity of CD34^+^ population, more vasculogenic fraction co-expressing another marker may exist in the mouse bone marrow CD34^+^ cells. The search for a truly selective EPC marker, marking “real” EPCs in combination with CD34, has thus to be continued.

## Materials and Methods

### Animals

C57BL6/J mice (male, 8–12 weeks old) were used for all experiments. Transgenic mice that constitutively expressβ-galactosidase (β-gal) under the control of the LacZ gene (B6;129S-Gt[ROSA]26Sor/J, male, 8–12 weeks old) were purchased from Jackson Laboratories (ME, USA) and used for in vivo experiments in which genetically labeled (positive for the LacZ gene) 50,000 SL, KL, KSL and CD34^+^ cells were systemically injected into wild type recipient mice after MI surgery.

### Preparation of KSL, KL, SL and CD34^+^ Cells

Bone marrow cells were harvested from hipbones, femurs, tibiae, shoulder bones, ulnas, vertebra and sternum of mice. BMMNCs were isolated from total bone marrow cells by subsequent purification over Histopaque gradients. BMMNCs of WT and ROSA26 mice were incubated with FITC-conjugated anti-CD34 antibody (BD Pharmingen) for 30 minutes at 4°C and CD34^+^ cells were further isolated using FACS Aria (BD Biosciences). For SL, KL and KSL cell isolation, BMMNCs were incubated with a cocktail of biotinylated monoclonal antibodies against lineage markers (BD IMag) for 15 minutes at 4°C. These cells were further incubated with streptavidin-conjugated magnetic nanoparticles (BD IMag) for 30 minutes. After depletion of lineage positive cells, lineage negative cells were subsequently incubated with PE-conjugated anti-Sca-1 antibody, (BD Pharmingen), APC-conjugated anti-c-Kit antibody (BD Pharmingen) and APC Cy7-conjugated streptavidin (BD Pharmingen) for 30 minutes at 4°C followed by isolation of the desired different sub-fractions KSL, KL and SL by FACS Aria (BD Biosciences).

### EPC Colony Forming Assay

The number of EPC colonies was assessed by EPC colony forming assay that we have developed recently [16], [22]. The KSL, KL, SL and CD34^+^ cells (500 cells/well) were cultured in methyl cellulose-containing medium M3236 (StemCell Technologies, Vancouver, Canada) with the following supplements in 6-well culture plates (Primaria, BD Falcon) for 7 days: 50 ng/ml of vascular endothelial growth factor (VEGF) (R&D Systems, MN), 20 ng/ml of stem cell factor (SCF) (KIRIN, Japan), 50 ng/ml of epidermal growth factor (EGF) (Wako, Japan), 20 ng/ml of interleukin-3 (KIRIN, Japan), 50 ng/ml of insulin-like growth factor-1 (IGF-1) (Wako, Japan), 50 ng/ml of basic fibroblast growth factor (bFGF) (Wako, Japan), 2 U/mL of heparin (AJINOMOTO, Japan) and 10% FBS. EPC-Colony Forming Units (CFUs) were identified as large-EPC-CFUs or small-EPC-CFUs by visual inspection with an inverted microscope under 40× magnification. Large-EPC-CFUs are mainly composed of spindle-shaped cells whereas small-EPC-CFUs are aggregates of small round cells. The endothelial phenotype of the EPC colonies was confirmed by their high up-take of acetylated low density lipoprotein (acLDL, Biomedical Technologies Inc, Stoughton, MA) and immunocytochemical positivity for isolectin B4 (Molecular Probes, CA). The total numbers of colonies with small-EPC-CFUs and large-EPC-CFUs were counted in the entire view fields of each culture plate and averaged.

### Collection and phenotypes of small and large colonies

After removal of methylcellulose containing floating and hematopoietic cells by cold PBS (1 ml/well) in 6-well plates, white spotty colonies (small colonies) were flushed gently with 1 ml of cold PBS for 3 times and collected. One ml of cold EDTA/PBS (5mmol/L) was then added to each well. The plates were then incubated for 5 minutes at 37°C and large colonies were collected. Small colonies and large colonies were incubated with PE-conjugated anti-CD19 antibody, PE-conjugated anti-CD3 antibody, PE-conjugated anti-CD14 antibody, PE-conjugated anti-CD41 antibody, PE-conjugated anti-CD31 antibody, PE-conjugated anti-Flk-1 antibody, PE-conjugated anti-Tie-2 antibody, PE-conjugated anti-Sca-1 antibody, APC-conjugated anti-VE-cadherin antibody, APC-conjugated anti-CD45 antibody, APC-conjugated anti-c-Kit antibody (BD Pharmingen), respectively, for 30 minutes at 4°C.After washing with PBS for 2 times, cells were analyzed by FACS Aria (BD Biosciences).

### EPC Culture Assay

Cellular adhesion capacity was evaluated as described previously [46]. Briefly, freshly isolated cells (10^5^ cells/well) were seeded in 20% fetal bovine serum (FBS)/EGM-2MV medium (Lonza) on a 8-well chamber slide coated with ProNectin F (Sigma), and cultured for 7 days at 37°C under 5%CO_2_. Each well was then washed 3 times with PBS and the attached cells were fixed with 4% paraformaldehyde (PFA)/phosphate buffer saline (PBS) and stained with DAPI. The attached cells were visualized under a fluorescence microscope and counted. The adhesive activity of each cell group was evaluated as the mean number of attached cells in each group.

### Immunocytochemistry

Cell samples were prepared for EPC culture assay as described above. Each cell-containing well was then washed three times with PBS and attached cells were briefly fixed with 4% PFA/PBS. For improved detection of intracellular proteins, cells were permeabilized by incubation in 0.1% Triton X-100/PBS solution for 5 min at room temperature (RT) followed by a three time rinse with PBS. Samples were blocked in antibody dilution buffer 2% BSA/PBS for 1 hour at RT. After removal of the blocking solution, primary antibodies were added: anti-VE-cadherin antibody (Santa Cruz, 1∶100), anti-vWF antibody (Chemicon, 1∶100), anti-Flk-1 antibody (Chemicon, 1∶100), anti-CD45 antibody (Abcam, 1∶200), anti-CD34 antibody (Abcam, 1∶50), anti-CD146 antibody (Abcam, 1∶100) in antibody dilution buffer at 4°C overnight. After washing three times with PBS for 5 min each, cells were incubated with secondary antibodies prepared at 1∶500 in antibody dilution buffer: Alexa 488 donkey anti-goat IgG, Alexa 488 goat anti-rabbit IgG and Alexa 488 goat anti-rat IgG (MP/Invitrogen) for 30 min at RT. After secondary antibodies were removed and cells were washed with PBS for three times, DAPI solution (Sigma, 1∶5000) was added and nuclei were stained for 10 min at RT. Mounting medium and a cover-slip were added to the glass slide followed by sealing samples with nail varnish before evaluation of the staining results under fluorescence microscopy.

### Quantitative Real-time RT-PCR

Total RNA was isolated from cell samples using RNeasy Mini kit (QIAGEN) according to the manufacturer's instructions. cDNA was synthesized using PrimeScript RT reagent Kit (TAKARA BIO Inc., Japan). For quantitative RT-PCR, the converted cDNA samples (2 µl) were amplified in triplicate in a final volume of 10 µl using SYBR Green Master Mix reagent (Applied Biosystems) and gene-specific primers with an ABI Prism 7700 RT-PCR machine (Applied Biosystems). Melting curve analysis was performed with Dissociation Curves software (Applied Biosystems) and the mean cycle threshold (Ct) values were used to calculate gene expression levels with normalization to mouse GAPDH.

### RT-PCR Primers

mGAPDH: forward: ACATCATCCCTGCATCCACT; reverse: CACATTGGGGGTAGGAACAC, mvWF: forward: ACGCCATCTCCAGATTCAAG, reverse: AAGCATCTCCCACAGCATTC, mVE-Cadherin: forward: TACTCAGCCCTGCTCTGGTT, reverse: GCTTGCAGAGGCTGTGTCTT, mFlk-1: forward: GGGTTTGGTTTTGGAAGGTT, reverse: CACGTAAGAGTCCGGAAGGA, mIntegrin beta 2: forward: GTACAGGCGCTTTGAGAAGG, reverse: TTTCAGCAAACTTGGGGTTC, mCXCR4: forward: TGCTGTGTGATGGTTTGTTTG, reverse: AAACCCCCAGCATTTCTACC.

### In Vitro Incorporation Assay

HUVECs and freshly isolated cells were used for tube formation assays as described previously [46]. CD34^+^, SL, KL, and KSL cells were labeled with DiI for 20 minutes. After washing with PBS, 2,000 of the DiI-labeled cells were mixed with 40,000 HUVECs in 100 µL of 10% FBS/EGM-2MV medium (Lonza) in order to evaluate the contribution of EPCs to EC-derived tube formation. One hundred µl of cell suspensions were applied to 100 µl of Matrigel (BD Biosciences) coated wells in an 8-well glass chamber slide (BD Falcon), and then incubated for 48 hours. The number of formed tubes and incorporated DiI-labeled cells were counted and averaged with the help of a computer assisted fluorescent microscope (OLYMPUS, Japan).

### Surgical Procedure

Institutional Animal Care and Use Committee in Institute of Biomedical Research and Innovation and RIKEN Center for Developmental Biology approved all the following research protocols (approval ID: AH21-01), including surgical procedures and animal care. Mice were anesthetized with 0.015 mg/kg of Avertin™ (Sigma). Myocardial infarction (MI) was induced as described previously [47]. Briefly, after the fourth to fifth intercostal space was opened, the heart was exteriorized and the pericardium was incised. Thereafter, the heart was held with a fine forceps, and MI was induced by ligation of the left anterior descending coronary artery at the just proximal site of the bifurcation of a diagonal branch with a 7-0 nylon suture followed by thorax closure. For in vivo study, 50,000 KSL, KL, SL and CD34^+^ cells together with PBS control were systemically injected 3 days after MI.

### Physiological Assessment of LV Function Using Echocardiography

Transthoracic echocardiography (SONOS 5500, Philips Medical Systems) was performed to evaluate LV function immediately before and 7 and 28 days after MI. After anesthesia with Avertin™ (Sigma), left ventricular end diastolic diameter (LVEDD), left ventricular end systolic diameter (LVESD), left ventricular ejection fraction (EF) and left ventricular fractional shortening (FS) were measured at the midpapillary muscle level. All procedures and analysis were performed by an experienced researcher who was blinded to treatment.

### Tissue Harvesting

For cell recruitment studies, MI-induced mouse hearts were harvested 24 hours after cell injection and prepared for frozen tissue sectioning after fixation with 4% PFA/PBS. For cell retention studies, MI-induced mice were anesthetized 28 days after surgery. Griffonia (Bandeiraea) Simplicifolia lectin 1 (Vector, 0.1 mg per mouse) was then injected systemically by direct cardiac puncture. Ten minutes later, the animals were euthanized, and hearts were harvested and prepared for paraffin tissue sectioning after fixation with 4% PFA/PBS.

### Morphometric Evaluation of Infarct Size

To elucidate the severity of myocardial fibrosis, Masson trichrome staining was performed on frozen sections from each tissue block, and the stained sections were used to measure the average ratio of fibrosis area to entire LV cross-sectional area (percent fibrosis area) and the average ratio of fibrosis length to entire internal LV circumference (percent fibrosis length).

### Immunofluorescence Staining

The sections were stained with rabbit anti-β-gal antibody (Cortex, 1∶1000) and antibody to Griffonia (Bandeiraea) Simplicifolia lectin 1 (Vector, 1∶100), then washed with PBS and stained with Alexa Fluor 488 rat anti-rabbit IgG and Alexa Fluor 546 rabbit anti-goat IgG (MP/Invitrogen, 1∶1000) at RT for one hour. Nuclei were counterstained with DAPI (Sigma, 1∶5000), and sections were mounted in aqueous mounting medium. Images were examined using a confoal microscope (OLYMPUS, Japan). To detect arterioles, the sections were stained with anti-α smooth muscle actin (SMA) antibody (Dako, 1∶250), then washed with PBS and stained with Alexa Fluor 594 donkey anti-mouse IgG2a (MP/Invitrogen, 1∶1000) at RT for one hour. Nuclei were counterstained with DAPI, and sections were mounted in aqueous mounting medium. Images were examined using a fluorescence microscope.

### Statistical analysis

All values were presented as mean ± SEM. Statistical analyses were performed with commercially available software (GraphPad Prism™, MDF Co Ltd, Japan). Comparisons between multiple groups were tested for significance via analysis of variance (ANOVA) followed by post-hoc testing with the Tukey's procedure. A *P* value less than 0.05 was considered statistically significant.

## Supporting Information

Figure S1 **CD34 positivity in KSL, KL and SL cells.** KSL cells were isolated from BMMNCs and further examined for CD34 expression by FACS. The analyzed data was shown as histogram. The percent of CD34 positivity was indicated in each histogram.
(TIF)

Figure S2 **Phenotypes of large and small colonies.** Large colonies and small colonies were collected separately after 7 days culture in methylcellulose medium. Phenotypes of large colonies (a) and small colonies (b) were analyzed by FACS. The analyzed data was shown as histogram. The percent of each positive population was indicated in each histogram.
(TIF)

Figure S3 **Assessment of CD45, CD146 and CD34 in cultured KSL, KL, SL, and CD34^+^ cells by immunocytostaining.** Freshly isolated cells were cultured in 20%FBS/EGM-2MV medium on vitronectin-coated 4 well-chamber slides for 7 days. Adherent cells were stained with CD45, CD146 and CD34. The assay was triplicated and demonstrated similar results.
(TIF)

Figure S4 **Endothelial marker expressions in freshly isolated mouse CD34+ cells.** CD34^+^ cells were isolated from mouse BMMNCs (10^5^ cells/mL) and further examined for CD31 (a), VE-cadherin (b), Flk-1 (c) and Tie-2 (d) expressions by FACS. The percent of each positive cell population was indicated in the contour plots.
(TIF)
