# Supplementary figures and images for: CD34+ Cells Represent Highly Functional Endothelial Progenitor Cells in Murine Bone Marrow
Source: PLoS One. 2011 May 31;6(5):e20219. doi: 10.1371/journal.pone.0020219 (PMC3105013; doi:10.1371/journal.pone.0020219)

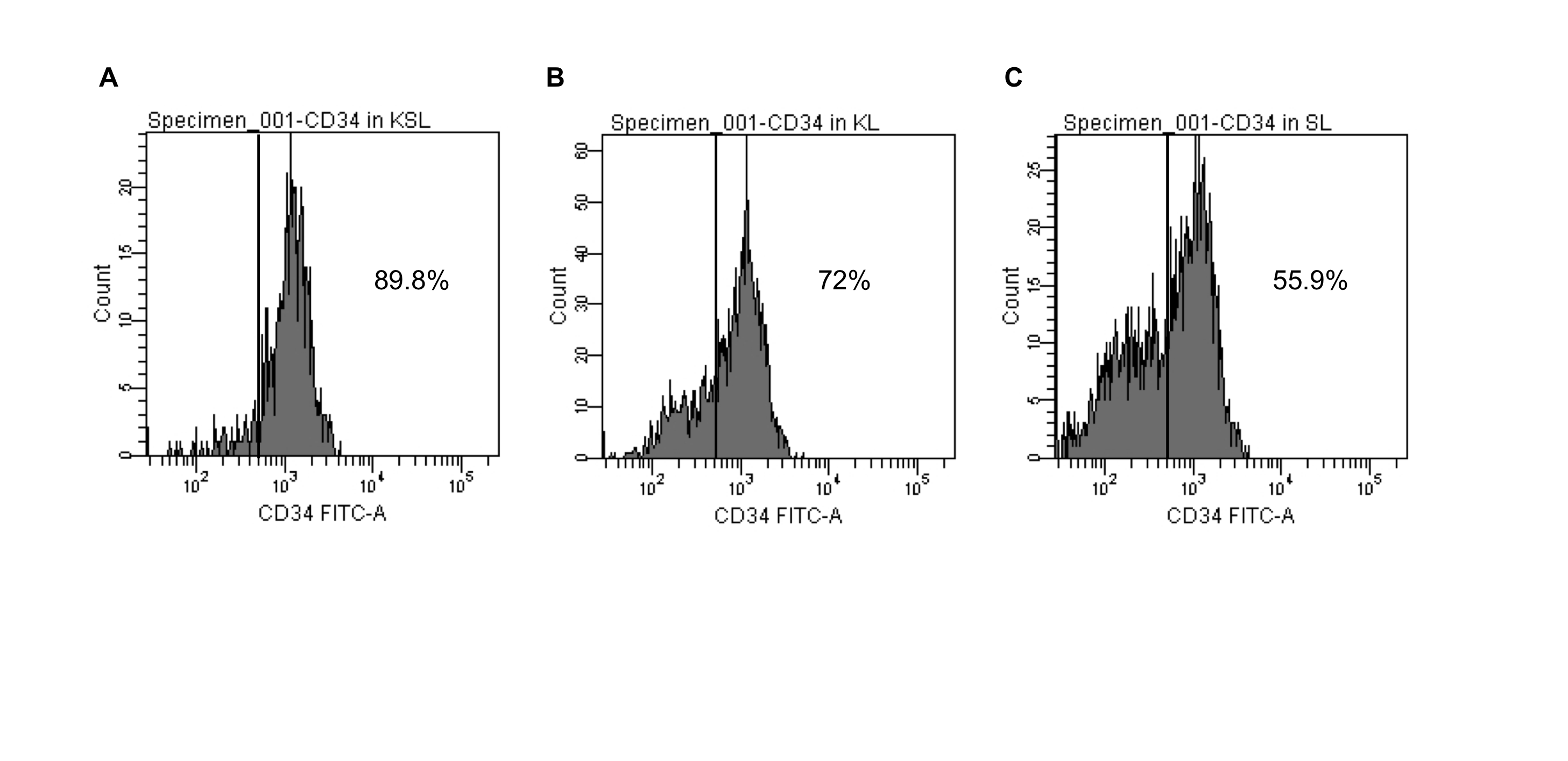

Supplement: Figure S1 — CD34 positivity in KSL, KL and SL cells. KSL cells were isolated from BMMNCs and further examined for CD34 expression by FACS. The analyzed data was shown as histogram. The percent of CD34 positivity was indicated in each histogram. (TIF) [file pone.0020219.s001.tif]

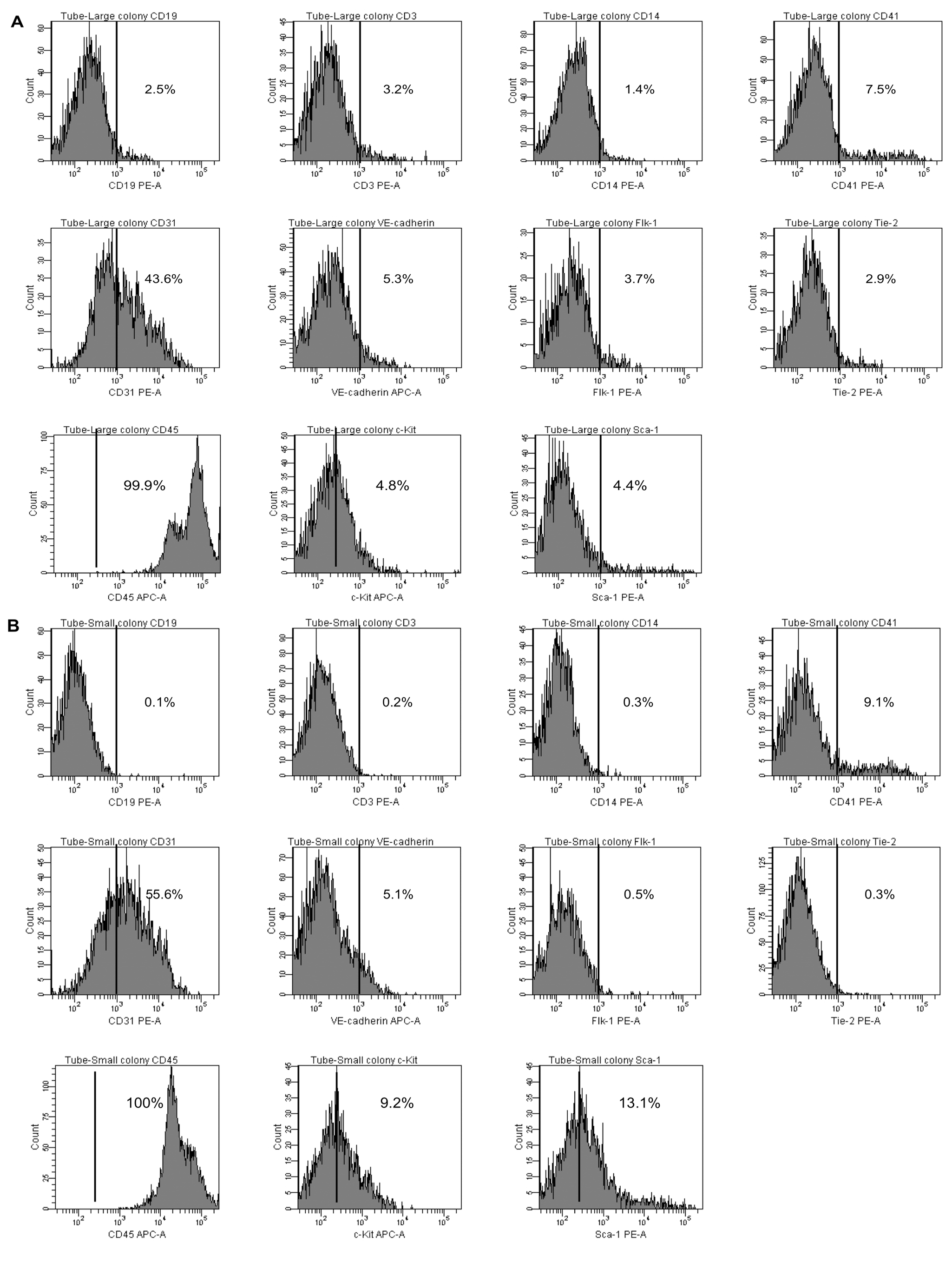

Supplement: Figure S2 — Phenotypes of large and small colonies. Large colonies and small colonies were collected separately after 7 days culture in methylcellulose medium. Phenotypes of large colonies (a) and small colonies (b) were analyzed by FACS. The analyzed data was shown as histogram. The percent of each positive population was indicated in each histogram. (TIF) [file pone.0020219.s002.tif]

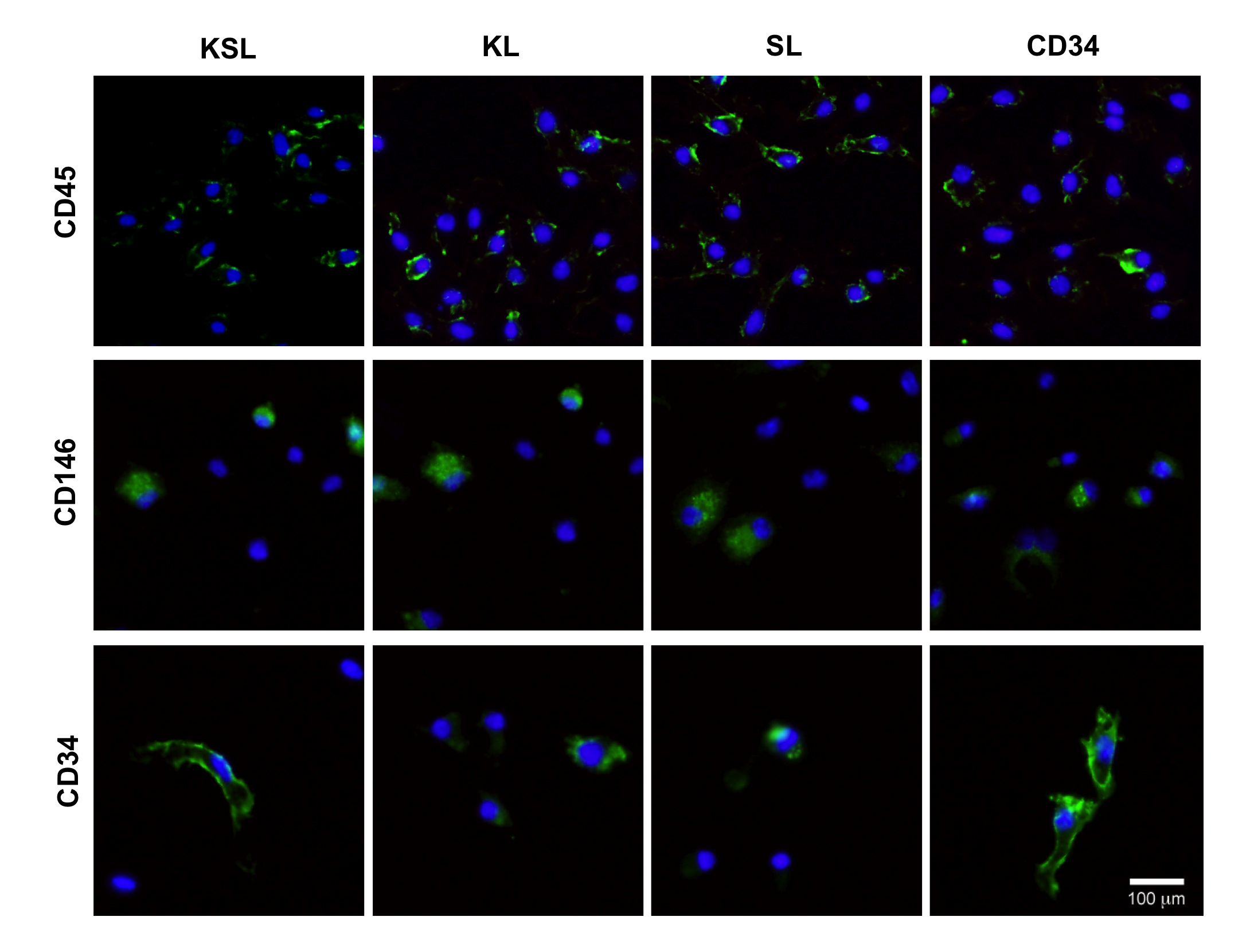

Supplement: Figure S3 — Assessment of CD45, CD146 and CD34 in cultured KSL, KL, SL, and CD34+ cells by immunocytostaining. Freshly isolated cells were cultured in 20%FBS/EGM-2MV medium on vitronectin-coated 4 well-chamber slides for 7 days. Adherent cells were stained with CD45, CD146 and CD34. The assay was triplicated and demonstrated similar results. (TIF) [file pone.0020219.s003.tif]

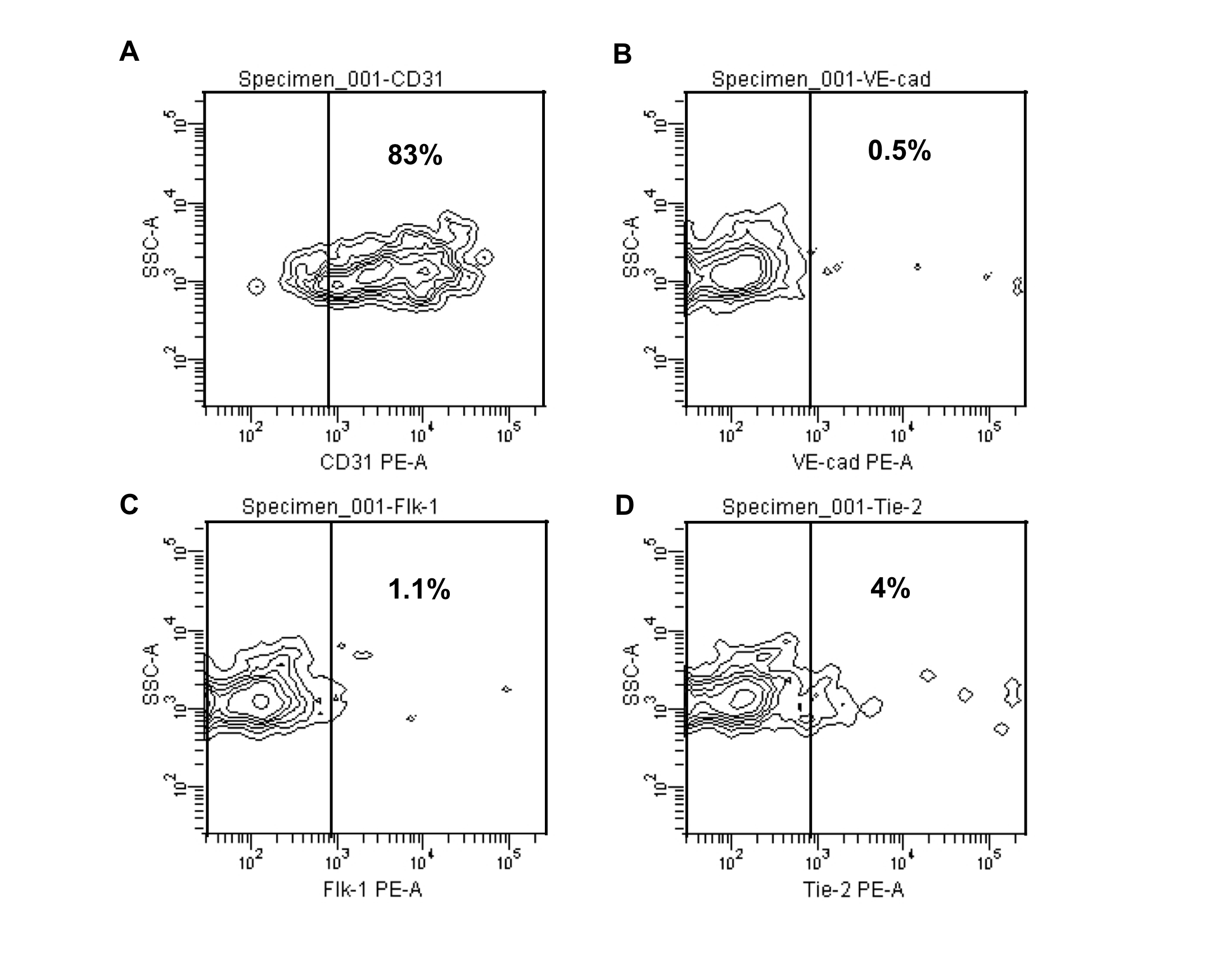

Supplement: Figure S4 — Endothelial marker expressions in freshly isolated mouse CD34+ cells. CD34+ cells were isolated from mouse BMMNCs (105 cells/mL) and further examined for CD31 (a), VE-cadherin (b), Flk-1 (c) and Tie-2 (d) expressions by FACS. The percent of each positive cell population was indicated in the contour plots. (TIF) [file pone.0020219.s004.tif]
